# Electrochemical Enzyme-Linked Immunosorbent Assay (ELISA) for α-Fetoprotein Based on Glucose Detection with Multienzyme-Nanoparticle Amplification

**DOI:** 10.3390/molecules181012675

**Published:** 2013-10-14

**Authors:** Qin-Lan Liu, Xiao-Hui Yan, Xiao-Mao Yin, Bo Situ, Han-Kun Zhou, Li Lin, Bo Li, Ning Gan, Lei Zheng

**Affiliations:** 1 Department of Laboratory Medicine, Nanfang Hospital, Southern Medical University, Guangzhou 510515, Guangdong, China; E-Mails: liuqinlan1206@163.com (Q.-L.L.); yinxiaomao@tom.com (X.-M.Y.); drstb@126.com (B.S.); yun.shally@163.com (L.L.); boli198777@163.com (B.L.); 2 Research Center of Clinical Medicine, Nanfang Hospital, Southern Medical University, Guangzhou 510515, Guangdong, China; E-Mail: gzyanxh@126.com; 3 The State Key Laboratory Base of Novel Functional Materials and Preparation Science, Faculty of Material Science and Chemical Engineering of Ningbo University, Ningbo 315211, China; E-Mail: zhouhankun@nimte.ac.cn

**Keywords:** glucoamylase, glucose biosensor, electrochemical enzyme-linked immunosorbent assay, starch, gold nanoparticles

## Abstract

Since glucose biosensors are one of the most popular and widely used point-of-care testing devices, a novel electrochemical enzyme-linked immunosorbent assay (ELISA) for protein biomarkers has been developed based on a glucose detection strategy. In this study, α-fetoprotein (AFP) was used as the target protein. An electrochemical ELISA system was constructed using anti-AFP antibodies immobilized on microwell plates as the capture antibody (Ab_1_) and multi-label bioconjugates as signal tracer. The bioconjugates were synthesized by attaching glucoamylase and the secondary anti-AFP antibodies (Ab_2_) to gold nanoparticles (AuNPs). After formation of the sandwich complex, the Ab_2_-glucoamylase-AuNPs conjugates converted starch into glucose in the presence of AFP. The concentration of AFP can be calculated based on the linear relation between AFP and glucose, the concentration of which can be detected by the glucose biosensor. When the AFP concentration ranged from 0.05 to 100 ng/mL, a linear calibration plot (i (µA) = 13.62033 − 2.86252 logCAFP (ng/mL), r = 0.99886) with a detection limit of 0.02 ng/mL was obtained under optimal conditions. The electrochemical ELISA developed in this work shows acceptable stability and reproducibility, and the assay for AFP spiked in human serum also shows good recovery (97.0%–104%). This new method could be applied for detecting any protein biomarker with the corresponding antibodies.

## 1. Introduction

Detection of protein biomarkers for cancer and other fatal human diseases plays a key role in aiding an early diagnosis and monitoring disease progression [1,2,3]. Biomarker detection technology has been applied for the diagnosis of a wide variety of conditions, including cancers, cardiac diseases, autoimmune diseases, and acute events such as stroke, cardiac ischemia, head injury and infectious diseases [4,5,6,7]. An effective detection method or device can not only be beneficial for early diagnosis and efficient treatment, but is also vital in preventing the spread of diseases and potentially eliminating the waste of resources on ineffective treatments [8,9]. Electrochemical biosensor devices have traditionally received a large share of the attention in diagnostic development [10]. Despite many technological advances in biosensor research and development and the introduction of many different products, very few devices are available for the public to use. Perhaps the most successful example of such a device is the glucose meter, which is currently one of the most widely used diagnostic devices in the world, as a result of the more than 30 years of development [11,12,13]. However, this widely used biosensor can only detect a single target, blood glucose. Future developments using glucose biosensors for protein biomarkers detection can potentially revolutionize the practice of medicine, with the ability to dramatically reduce healthcare costs. In this aspect, glucose biosensors could serve as general detectors used for both glucose and protein targets.

The major challenge in using glucose biosensors for targets beyond glucose is to find a way that links the detection of glucose to that of the desired protein targets. Glucoamylase can provide this link. Glucoamylase is a form of amylase that cleaves the last α-1-4-glycosidic linkages at the non-reducing end of amylopectin and the α-1-6-glycosidic linkages to yield glucose. Moreover, compared with other enzymes used in immunosensors, such as alkaline phosphatase (ALP) [14,15,16], horseradish peroxidase (HRP) [17,18,19,20,21,22] and glucose oxidase (GOD) [23], glucoamylase is cost effective. Although glucoamylase can efficiently hydrolyze starch into glucose, there is need to amplify the signal in order to enhance detection sensitivity. Among all kinds of signal amplification approaches, the most popular strategy is to employ a new matrix for labeling more enzymes loaded on the detection antibody or antigen. Signal amplification based on biofunctional nanomaterials has recently attracted considerable attention due to their outstanding electronic and biocompatible performance. Gold nanoparticles (AuNPs) have widely been used as labels for analytical signal amplification and biomedical purposes for many years [24,25,26,27]. AuNPs have such advantages as rapid and easy synthesis, narrow size distribution, efficient surface modification by thiols or other bioligands, and their desirable biocompatibility. Furthermore, AuNPs can be easily conjugated with biomolecules and retain the biochemical activity of the tagged biomolecules, leading AuNPs to be excellent transducers for several biorecognition applications [26,27,28,29,30,31].

**Scheme 1:**
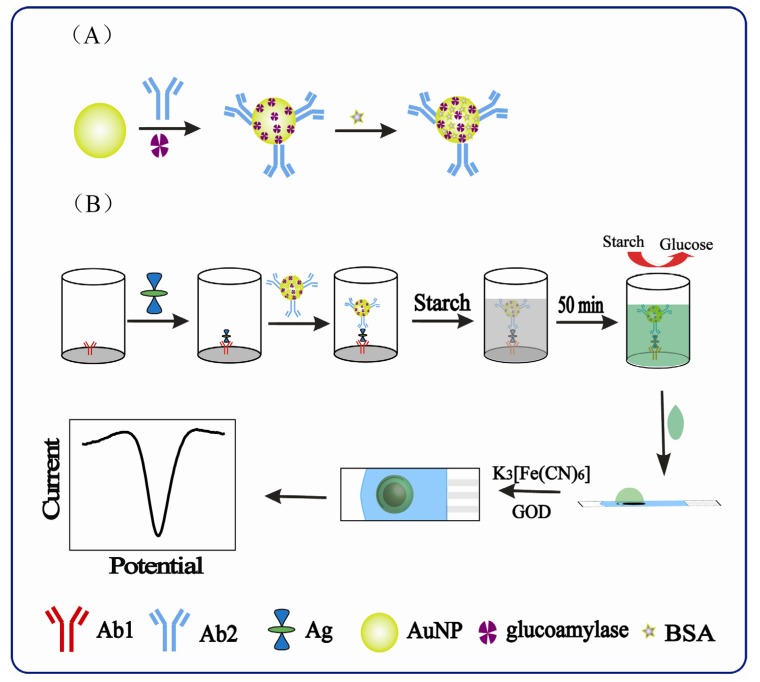
Schematic representation of (**A**) the preparation of bioconjugates, and (**B**) ELISA and measurement procedure.

In this study, we take advantage of a highly developed and commercially successful technology, the electrochemical quantification of glucose in blood, for protein biomarkers detection. This novel strategy, electrochemical ELISA for protein biomarkers based on glucose detection, used AFP as a model protein. Glucoamylase was used as a labeled molecule which acted as a link between glucose and AFP by converting starch into glucose. Enhanced sensitivity was achieved by using AuNPs as a nanocarrier to attach glucoamylase and signal antibody at high ratio. The processes used to perform the ELISA and electrochemical detection are outlined in Scheme 1B. 

The procedure involved three steps: immunoreactions, production of glucose and detection of glucose. The sandwich immunoreactions were carried out in a microwell to obtain the sandwich immunocomplex Ab_1_/AFP/Ab_2_-glucoamylase-AuNPs in the presence of AFP. Glucose was generated after adding starch into the microwell after obtaining the sandwich immunocomplex. Starch is a carbohydrate consisting of a large number of glucose units joined by glycosidic bonds, which can be converted into glucose by glucoamylase. These two steps were carried out in microwell plates. While, the concentration of glucose can be easily detected by glucose biosensors, which were developed by adding glucose oxidase (GOD) and K_3_[Fe(CN)_6_] to screen-printed carbon electrodes (SPCEs) after dropping glucose solution onto the electrodes. Then the concentration of AFP in samples can be calculated via the linear relation between AFP and glucose, the final product of the ELISA.

## 2. Results and Discussion

### 2.1. Characterization of Ab_2_-AuNPs-Glucoamylase Conjugates

**Figure 1:**
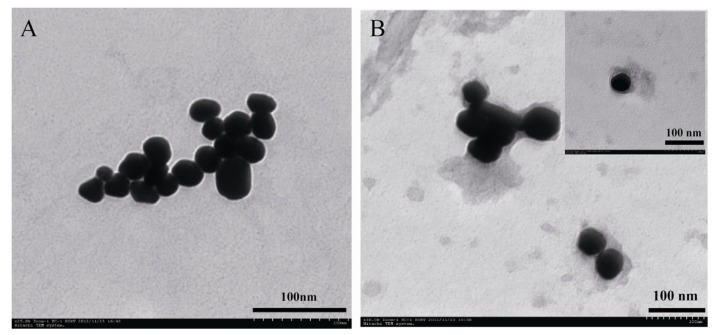
Transmission electron micrographs images of AuNPs (**A**) and Ab_2_/glucoamylase/AuNPs (**B**).

The steps for labeling Ab_2_ are illustrated in Scheme 1A. AuNPs and conjugates were characterized by TEM with the images shown in Figure 1. Figure 1A demonstrates the AuNPs to be dispersible and stable in solution. The average diameter of the nanoparticles was approximately 50 nm. Through the interaction between amine or SH groups in protein and AuNPs, the antibody and enzyme could be easily loaded on the surface of AuNPs to form conjugates. After coating the AuNPs with Ab_2_, glucoamylase and BSA, a layer of electron-lucent substance around the black AuNPs was observed (Figure 1B), this confirmed the attachment of proteins on the surface of the nanoparticles. The sensitivity of the detection was enhanced and amplified by increasing the amount of enzymes on the surface of AuNPs (Figure 1).

**Figure 2:**
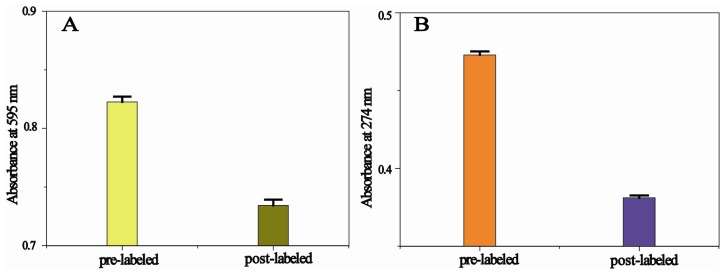
(**A**) Absorbance readings taken at 595 nm, following Coomassie Brilliant Blue G-250 staining, to observe the amount of Ab_2_ in pre-labeled and post-labeled Ab_2_ solutions. (**B**) Absorbance readings taken in the UV-Vis spectra (274 nm), for the detection of binding of glucoamylase to AuNPs.

Coomassie Brilliant Blue G-250 staining was performed to observe the concentration of Ab_2_ in pre-labeled and post-labeled clear supernatant (Figure 2A). Coomassie Brilliant Blue G-250 can be used to quantify microgram quantities of protein in a solution, whereby the optical absorbance of the solution is measured at a wavelength of 595 nm [32]. As illustrated in Figure 2A, the absorbance values at 595 nm were 0.82 and 0.73 for pre-labeled and post-labeled Ab_2_ solutions, respectively. This decrease proved the attachment of Ab_2_ onto AuNPs. The conjugation process was further recorded by UV-vis spectroscopy as shown in Figure 2B. The clear supernatant obtained from pre-labeled and post-labeled glucoamylase solutions was carried out with UV-Vis spectra. The absorbance at 274 nm reduced from 0.47 to 0.38 (Figure 2B), indicating successful binding of glucoamylase to AuNPs. When Ab_2_ and glucoamylase were adsorbed onto AuNPs, a reduced absorbance value for two labeled clear supernatants was observed (Figure 2), which proved the attachment of Ab_2_ and glucoamylase onto AuNPs.

### 2.2. Optimization of Experimental Parameters

**Figure 3:**
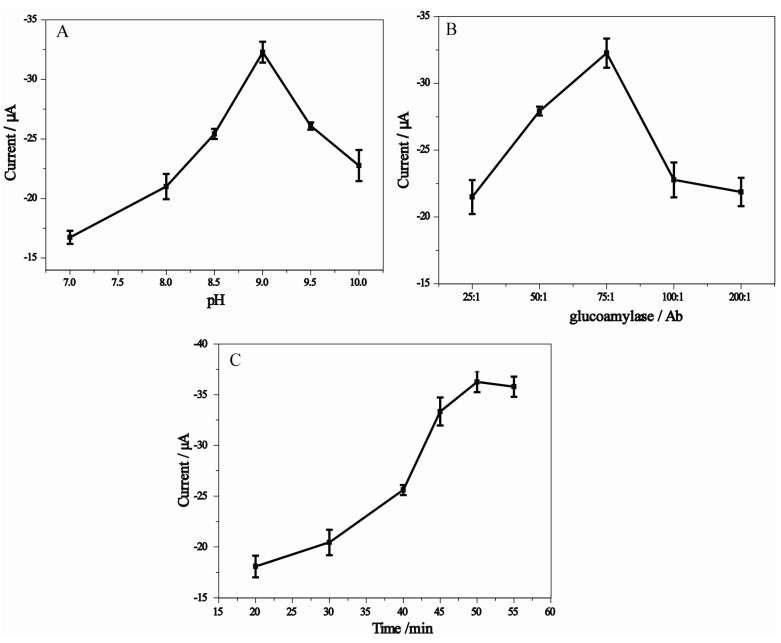
(**A**) Optimal pH for the conjugation of glucoamylase and Ab_2_ to AuNPs. (**B**) Effect of glucoamylase/Ab_2_ ratio on response current. (**C**) Effect of incubation time on the DPV peak current.

In order to obtain the best analytical performance for AFP detection, experimental conditions were optimized. The pH played a vital role in preparation of anti-AFP-AuNPs-glucoamylase conjugate. As can be seen in Figure 3A, the highest assay sensitivity was achieved when the conjugate prepared at pH 9.0 was used. 

This pH is, therefore, the optimal for conjugation, ensuring the highest level of gold surface coverage by the antibody and glucoamylase. The ratio of glucoamylase and Ab_2_ (glucoamylase/Ab_2_) is one of the most important factors affecting the response signal, owing to the co-immobilization of enzymes and antibodies on the gold nanocarrier. As shown in Figure 3B, the electrocatalytic current increases with increasing ratio of glucoamylase/Ab_2_, and the maximum response is achieved at a ratio of 75:1. The increase in glucoamylase/Ab_2_ ratio could increase the total amount of glucoamylase loaded per gold nanoparticle, which is expected to improve the response amplification. However, reducing the amount of Ab_2_ in the bioconjugates may decrease the immunocoupling efficiency in the captured Ab-antigen in the microwell plates, which may result in a decreased response. Therefore, a glucoamylase/Ab_2_ ratio of 75:1 is selected as the optimal condition to prepare the Ab_2_-AuNPs-glucoamylase conjugates.

Incubation time with the bioconjugates is an important parameter affecting the analytical performance of the immunoassays, which was carried out using the described electrochemical conditions. The DPV peak current for AFP increased with increasing incubation time and tended to a constant value after an incubation time of 50 min (Figure 3C), which shows a saturated binding between the analytes and the antibodies. Therefore, an incubation time of 50 min was selected for the sandwich-type immunoassay.

### 2.3. DPV Response of the Glucose Biosensor to AFP Concentration

**Figure 4:**
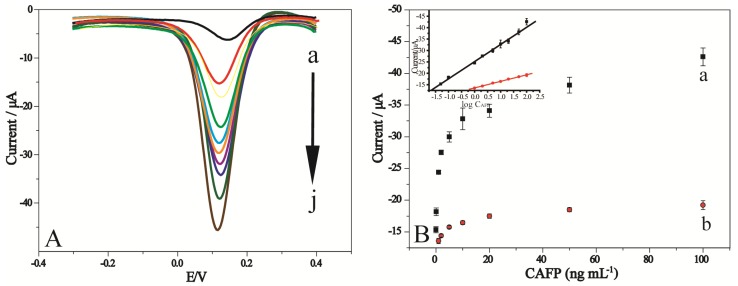
(**A**) DPV curves acquired at different concentration of glucose produced by Ab_1_/AFP/Ab_2_-AuNPs-glucoamylase, incubated with (a) 0, (b) 0.05, (c) 0.1, (d) 1, (e) 2, (f) 5, (g) 10, (h) 20, (i) 50, (j) 100 ng/mL AFP antigen. (**B**) Calibration curves for detecting AFP antigen were obtained using Ab_2_-glucoamylase (a) and Ab_2_-AuNPs-glucoamylase (b) as the signal tag, respectively. Inset: linear relationship between the amperometric oxidation current response and the logarithm of AFP concentrations.

Under optimum conditions, the DPV peak currents of glucose detected by the glucose biosensor increased with increasing concentration of AFP in the incubated solution. Figure 4A shows typical DPV curves corresponding to glucose produced by sandwich-type immunocomplexes Ab_1_/AFP/Ab_2_-glucoamylase-AuNPs hydrolyzed starch in microwell plates for AFP concentrations ranging from 0 to 100 ng mL^−1^.

The results suggested that the AFP concentration could be determined with the glucose-detection-based electrochemical ELISA method. Furthermore, these results also demonstrated the success of a novel electrochemical immunoassay based on glucoamylase mediator. The standard calibration curve for AFP detection is illustrated in Figure 4B. The electrochemical signal increased linearly with the logarithm of AFP concentrations in the range from 0.05 to 100 ng/mL (r = 0.99886), and the detection limit was 0.02 ng/mL with a signal-to-noise ratio of 3. According to the linear equation, AFP concentration can be detected quantitatively. Higher AFP levels could be detected by an appropriate dilution with pH 7.0 PBS.

### 2.4. Comparison of Electrochemical Responses

One major advantage in using nanoparticles is the possibility to control and tailor their properties to meet the needs of specific applications [33]. The prepared standard solutions of AFP were analyzed using the electrochemical ELISA procedure, with and without the use of AuNPs enhancers. The electrochemical ELISA procedure optimized in this work used Ab_2_-AuNPs-glucoamylase bioconjugates as a signal tag. As shown in Figure 4B when gold nanobioconjugates were used, higher signals were recorded, resulting in a more sensitive assay. This was shown in the inset which exhibits a less steep decline with a linear response from 1 to 100 ng/mL. These results indicated that using Ab_2_-AuNPs-glucoamylase instead of Ab_2_-glucoamylase as a signal tag enhanced the sensitivity of this method. We believe that this result demonstrates that AuNPs act successfully as a sort of “concentrator” for the signal antibody onto the antigen binding sites.

### 2.5. Selectivity and Repeatability

**Figure 5:**
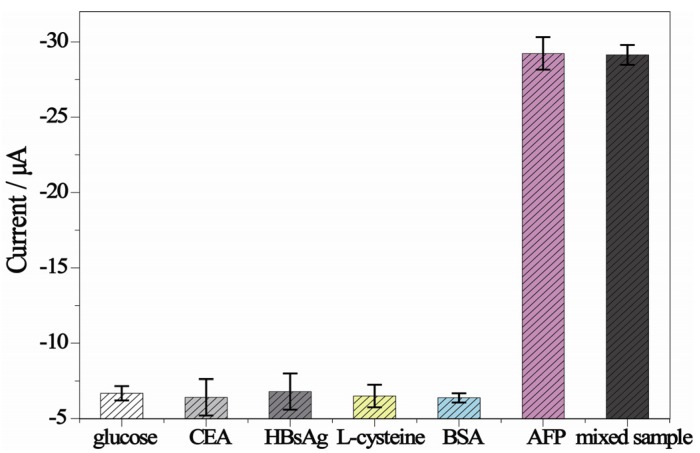
Selectivity of the electrochemical ELISA system to AFP (5 ng/mL) by comparing it to the interfering protein: glucose (50 ng/mL), carcinoembryonic antigen (CEA, 50 ng/mL), hepatitis B virus surface antigen (HBsAg, 50 ng/mL), L-cysteine (50 ng/mL), bovine serum albumin (BSA, 50 ng/mL), and the mixed sample containing 5 ng/mL AFP, 50 ng/mL glucose, 50 ng/mL CEA, 50 ng/mL HBsAg, 50 ng/mL L-cysteine and 50 ng/mL BSA.

Selective determination of target analytes played a vital role in analyzing biological samples. To investigate the specificity of the electrochemical ELISA system, we challenged the system with other biomarkers including glucose, CEA, HBsAg, L-cysteine, and BSA. As can be seen from Figure 5, the current response of the proposed method showed no remarkable change in AFP mixed with interfering agents compared to AFP only. On the contrary, low current responses were exhibited by replacing AFP with glucose, CEA, HBsAg, L-cysteine, and BSA in the solution. These results clearly indicated the high specificity of the electrochemical ELISA method.

Reproducibility of the immunoassay for AFP was investigated with intra- and inter-assay precision. Intra-assay precision of the immunoassay was evaluated by assaying one level of AFP for five similar measurements on one SPCE. Inter-assay precision was estimated by determining one AFP level with five SPCEs. The GOD and K_3_[Fe(CN)_6_] were not immobilized on SPCEs but added to the detection solution, so we can achieve stable results in the same SPCE and the SPCE can be used more than 40 times. The intra- and inter-assay variation coefficients obtained from 50 ng/mL of AFP were 4.4% and 8.2%, respectively. These results demonstrated that the electrochemical ELISA method possessed acceptable reproducibility.

### 2.6. Application of Electrochemical ELISA in Human Serum Samples

The applicability of the proposed method in the clinical field was investigated by analyzing several human serum samples. Table 1 shows the analytical results of AFP in five independent human serum samples. It was found that the recoveries were in the range of 97.0%–104% (Table 1), which was satisfactory for quantitative assays performed in biological samples.

**Table 1:** Recovery of AFP in human serum samples.

Sample	AFP found (ng/mL)	AFP added (ng/mL)	Total AFP detected (ng/mL)	Recovery (%)	RSD (n = 5)
1	0	0.5	0.52	104	7.50%
2	1.40	1.00	2.46	102.5	6.30%
3	1.90	10.00	11.54	97.0	4.90%
4	1.20	20.00	22.01	103.8	5.10%
5	2.00	50.00	50.83	97.8	3.50%

## 3. Experimental

### 3.1. Materials and Reagents

Standard AFP solutions from 5 to 200 ng/mL and capture antibody-coated (Ab_1_) microwell plates were purchased from Biocell Company (Zhengzhou, China). Mouse monoclonal signal anti-AFP antibody (Ab_2_ or detection antibody, 13.34 mg/mL) was supplied by Biocell Company (Zhengzhou, China). Chloroauric acid (HAuCl_4_·4H_2_O) and bovine serum albumin (BSA, 96%–99%) were purchased from Sinopharm Group Chem. Re. Co., Ltd. (Shanghai, China). All other chemicals were of analytical grade and used as received. GOD (EC 1.1.3.4, 100–250 U/mg, type x-p from *Aspergillus niger*) was purchased from Sigma (St. Louis, MO, USA). Glucoamylase was purchased from Aobox Company (Beijing, China).

### 3.2. Apparatus

Electrochemical measurements were performed using an electrochemical detector (CH Instruments 660d, Shanghai, China). Transmission electron microscope (TEM) images were obtained on an H-7650 transmission scanning electron microanalyzer (Hitachi Instruments, Tokyo, Japan) equipped with a CCD camera. The optical density at 595 nm was measured using a microtiter plate reader (SpectraMax M5, Molecular Devices, Sunnyvale, CA, USA). UV-Vis spectra were recorded using a Thermo Scientific NanoDrop 2000/2000c spectrophotometer (Thermo, Waltham, MA, USA). Screen-printed carbon electrodes (SPCEs, 4 mm diameter) were purchased from DropSens Corporation (Lianera, Spain), and consisted of a carbon working electrode, a carbon auxiliary electrode, and an Ag/AgCl reference electrode.

### 3.3. Synthesis of Colloidal Gold Particles

Gold nanoparticles were synthesized by reducing chloroauric acid with sodium citrate [34]. One hundred mL of 0.048% HAuCl_4_ solution was boiled with vigorous stirring, and 6 mL of a 0.5% sodium citrate solution was then added quickly to the boiling solution. When the solution turned deep red, indicating the formation of gold nanoparticles, the solution was cooled down. The synthesized AuNPs were characterized using TEM (Figure 1A) to measure the size and morphology.

### 3.4. Procedures for the Synthesis of the Ab_2_-AuNPs-Glucoamylase Conjugates

The preparation procedures of the Ab_2_-AuNPs-glucoamylase conjugates were illustrated in Scheme 1A. To synthesize Ab_2_-AuNPs-glucoamylase conjugates, Ab_2_ and glucoamylase were attached to gold nanoparticles with a reaction mixture of 75:1 glucoamylase/Ab_2_ weight ratio. Briefly, Ab_2_ (13.34 mg/mL) was added to 1 mL of AuNPs, which were previously adjusted to pH 9.0 with 0.5 M NaOH, followed by incubation at 4 °C for 5 min with slow stirring. Next, 200 mg mL^−1^ glucoamylase was dropped into the suspension and the resulting solution was gently stirred at 4 °C for 6 h. Then 0.2 mL blocking solution was dropped into the suspension and the resulting solution was gently stirred at 4 °C for 2 h. Finally, the reaction mixture was separated by centrifuging at 10,650 g for 3 min, and the supernatant was discarded. The resulting mixture was washed with ultrapure water for three or four times and later dispersed in 1 mL 1% BSA and stored at 4 °C. The bioconjugates thus obtained were diluted five times with ultrapure water before use.

### 3.5. Measurement Procedure

Scheme 1B was a schematic of the whole assay steps used in this work. The immunoreaction was performed in microwell plates. To carry out the immunoreaction and electrochemical measurements, one hundred microliters of AFP standard solution or serum sample of the desired concentration was dropped into the microwell plate coated with capture antibody, and incubated sequentially at 37 °C for 30 min. After being washed with washing buffer, 100 µL of glucoamylase-labeled detecting antibody or Ab_2_-AuNPs-glucoamylase conjugates were dropped into each well and incubated for 50 min at 37 °C, followed by five washing steps with PBS. Subsequently, excess antibody was removed with washing buffer. Afterward, 40 µL of 20 mg/mL pH 4.0 starch was dropped into the well and followed by incubation at 45 °C for 30 min. Finally, the solution containing an amount of glucose was ready for detection.

The reaction mixture (30 µL) was then dropped on to the SPCE reservoir area to cover the working, counter, and reference electrodes. To avoid the possibility of contamination from the working electrodes, oxygen plasma treatments were carried out on the SPCEs first. Differential pulse voltammetry (DPV) was used as the electrochemical measurements in this study. The electrochemical signal of glucose was produced by oxidizing glucose by GOD. The DPV measurements were carried out by scanning at 100 mV/s between −0.3 V and +0.4 V. Its peak current was collected and registered as signal of glucose, which was proportional to the concentration of AFP in samples. The detection principle of ELISA and the electrochemical assay were outlined in Scheme 1B.

## 4. Conclusions

A successful approach for the development of an electrochemical immunoassay for AFP detection has been reported in this study. This method is based on transforming the concentration of protein biomarkers in samples into that of glucose produced from glucoamylase conjugates hydrolysis of starch. Due to the concentration of the glucoamylase conjugates bound to the surface of microwell plates correlating to the concentration of the targets in the sample, the concentration of glucose produced from starch hydrolysis by glucoamylase conjugates can be used to calculate the concentration of AFP in the sample after measurement using glucose biosensors. The proposed electrochemical ELISA shows excellent performance for AFP within a wide linear range with low detection limit and acceptable stability, reproducibility, and accuracy. Antibodies for a wide range of protein biomarkers are readily available, allowing the methodology shown here to be applied as a general platform for many other disease biomarkers.
